# Measurement of Hand/Handrim Grip Forces in Two Different One Arm Drive Wheelchairs

**DOI:** 10.1155/2014/509898

**Published:** 2014-06-19

**Authors:** Anne Mandy, Lucy Redhead, Jon Michaelis

**Affiliations:** ^1^Brighton Doctoral College, University of Brighton, Aldro 49 Darley Road, Sussex, Eastbourne BN20 7UR, UK; ^2^University of Brighton, Robert Dodd, 49 Darley Road, Sussex, Eastbourne BN20 7UR, UK; ^3^Neater Solutions, 12 Burlington Road, Derbyshire, Buxton SK17 9AL, UK

## Abstract

*Purpose*. The aim of this study was to explore the total and regional grip forces in the hand when propelling two different manual one arm drive wheelchairs: the Neater Uni-wheelchair (NUW) and a foot steered Action3 wheelchair. *Methods*. 17 nondisabled users were randomly assigned to each wheelchair to drive around an indoor obstacle course. The *Grip*, a multiple sensor system taking continuous measurement of handgrip force, was attached to the propelling hand. Total grip force in each region of the hand and total grip force across the whole hand were calculated per user per wheelchair. *Results*. The Action3 with foot steering only generated significantly greater total grip force in straight running compared to the NUW and also in the fingers and thumb in straight running. *Conclusions*. The results suggest that the Action3 with foot steering generated greater grip forces which may infer a greater potential for repetitive strain injury in the upper limb. Further work is required to explore whether the difference in grip force is of clinical significance in a disabled population.

## 1. Introduction

Manual wheelchair propulsion is known to be an inefficient means of ambulation which has been associated with a high prevalence of upper limb injuries [1, 2]. Such injuries are thought to occur from a combination of repetitive movements, upper limb weakness, and inefficient propulsive technique [3, 4]. Hemiplegic users are particularly vulnerable to upper limb injury and pain [5, 6] because of being reliant on only one arm for propulsion. Moreover, the action of manual propulsion necessitates that the hand exerts repetitive forces to the handrim [6, 7] in order that the hand/handrim coupling be stable to accommodate the transfer of the forces from the shoulder and arm muscles onto the handrim [7]. This repetitive grip action may contribute to the development of upper limb repetitive strain injury [6, 8]. In a standard wheelchair, the handrim design has also been suggested to be one of the factors responsible for the low mechanical efficiency in manual propulsion [9].

**Figure 1:**
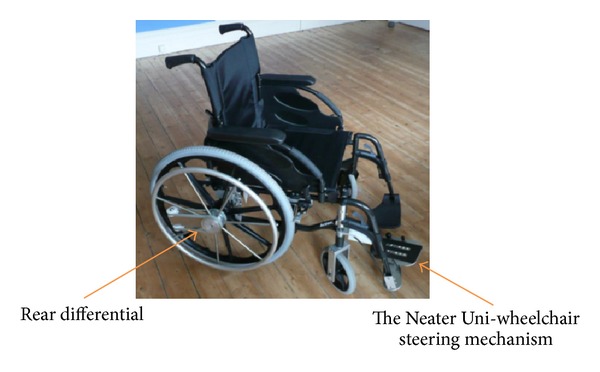
The Neater Uni-wheelchair.

**Figure 2:**
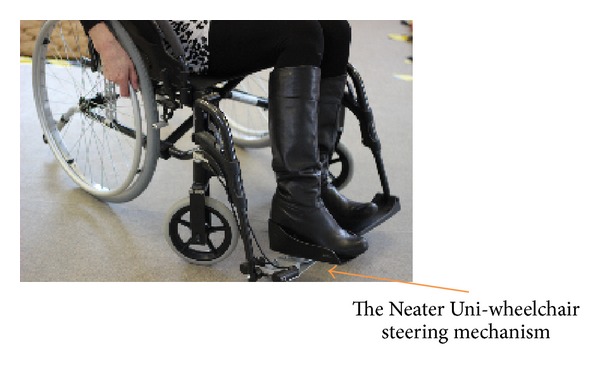
The foot steered standard Action3 wheelchair.

There is currently very little choice of one arm drive wheelchairs. The most commonly prescribed include the one arm ratchet arm or lever-drive mechanism and the dual handrim mechanism. There are deficiencies associated with both of these designs, particularly with respect to the user interface. For a large number of users, the overall ergonomics of operation is not efficient and recent research has suggested that neither may be suitable for hemiplegic users [10]. A recent alternative to these has been the development of the Neater Uni-wheelchair (NUW) which has been designed specifically for hemiplegic users in response to the identified problems associated with the other two market alternatives. The NUW is an Action3 wheelchair to which a rear wheel differential and front wheel steering kit are attached. These features have been described in detail in a range of studies comparing it to both the dual handrim and lever-drive alternatives [11–15]. The differential enables a single pushrim to propel both rear wheels equally resulting in the wheelchair moving in a straight line. The steering mechanism is attached to one foot plate and operates independently of the drive mechanism. Steering is intuitive: rotating the foot to the right turns the wheelchair to the right; rotate the foot to the left and chair turns left. In addition the kits can be attached to either side for use by either right- or left-handed users (insert Figures 1 and 2) and can include both components or only the steering mechanism. The body of work to date, comparing the NUW to existing provision, suggests that the NUW is ergonomically more efficient to drive and preferred by users in a laboratory setting [11, 12], in their own homes [12, 13], and in simulated activities of daily living setting [14]. These studies suggested that NUW could meet the unmet needs of the hemiplegic user group and provide them with additional choice in their wheelchair provision. However, there is no research exploring grip action in different one arm drive wheelchairs.

Wheelchair propulsion necessitates the repetitive use of the upper limb joints and muscles which have been linked to repetitive injuries in manual wheelchair users [7]. A recent study, measuring shoulder muscle activity using EMG in one arm drive wheelchairs, has shown an increase in activity levels when propelling the Action3 wheelchair when compared to the NUW [16]. These changes in muscle activity may be linked to the incidence of repetitive injuries in wheelchair user. Repetitive injuries have also been associated with grip action during manual wheelchair propulsion. Early research surrounding hand function in wheelchair propulsion has focused on distinguishing between power grip and precision grip [17]. Whilst these positions have been explored extensively in terms of biomechanic, ergonomic, and functional differences, the findings are equivocal. Chao et al. [18] and Cooney and Chao [19] suggested that a strong pincer grip overloads forearm tendons more than a power grip. Conversely Edgren et al. [20] suggest a correlation between the tension of the long finger tendons and handgrip diameter suggesting that the optimization of the handrim diameter is important in reducing effort. This was further endorsed by Peebles and Norris [21] who suggested that grip force in all age groups can be affected by the handle diameter, position, and movement direction. The measurement of local force in the hand is an important aspect to be investigated in any hand tool evaluation process. Instrumented gloves with force sensor resistors (FSR) have been used to measure mechanical loads on the hand's surface during a prehensile activity and have been used for biomechanical evaluation studies [22, 23]. More recent work by Mastalerz et al. [24], using the* Grip* Force Measurement System, explored the maximal strength capabilities of the hand, regarding the grip handle diameter and position, among young men and women but failed to identify an optimal handle diameter generating the maximal handgrip force. Whilst these studies have provided valuable information on kinetics of grip function, the understanding of the interaction between hand surface and handrim of one arm drive wheelchairs remains unclear. Medola et al. [25] measured pressure in different components of the hand, including the thumb, palm, and finger tips, during manual wheelchair propulsion using a similar instrumented system to the* Grip*. The results showed that the hands hold the rim with the distal phalanges of the fingers, concentrating higher pressures on these areas. This could indicate increased loading in the flexor tendons of the forearm which may contribute to the development of overuse injury [19]. The research to date has only explored handrim grip force in standard wheelchairs and not one arm drive wheelchairs. The aim of this study was to explore the total and regional grip forces within the hand exerted at the hand/handrim interface in two different manual one arm drive wheelchairs.

The research hypothesis was as follows. There will be differences in total and regional grip forces at the hand/handrim interface when propelling different manual one arm drive wheelchairs.

## 2. Methods

Ethical approval was sought and obtained from the University of Brighton Research Ethics Committee for the study.

Participants were recruited from the University of Brighton Campus using posters. The inclusion criteria were willingness to participate, no cardiac or respiratory disorder, no functional impairment, hand size to fit medium size vinyl glove, right-hand being dominant and being within the height and weight restrictions of 163–185 cm height and 54–90 kg weight. Exclusion criteria was inability to learn how to propel the wheelchair safely. Participants were provided with an information sheet prior to being recruited into the study to enable them to make an informed decision concerning their involvement. All participants who wished to take part completed a health declaration sheet and informed consent sheet.

The study was designed as a controlled, same subject study that measured grip force generated by each user during propulsion in two different manual one arm drive wheelchairs. The two wheelchairs, as described in the introduction, were

1. an Action3 wheelchair with Neater Uni-wheelchair kits applied which included the differential in the rear wheel and the foot steering mechanism (Figure 1);
2. an Action3 wheelchair with only the Neater Uni-footplate steering and a standard handrim for propulsion (Figure 2).

The drive wheels were the same size and diameter in both wheelchairs. There was no difference in weight between the wheelchairs.

## 3. Materials

### 3.1. The* Grip* Force Measurement System

The data being measured were grip force at the hand/handrim interface using the Tekscan* Grip* Force Measurement System. This is a portable interface force mapping system which records grip force distribution under the contact area. Tekscan force and pressure measurement systems have been demonstrated to generate reliable and valid data [26].

**Figure 3:**
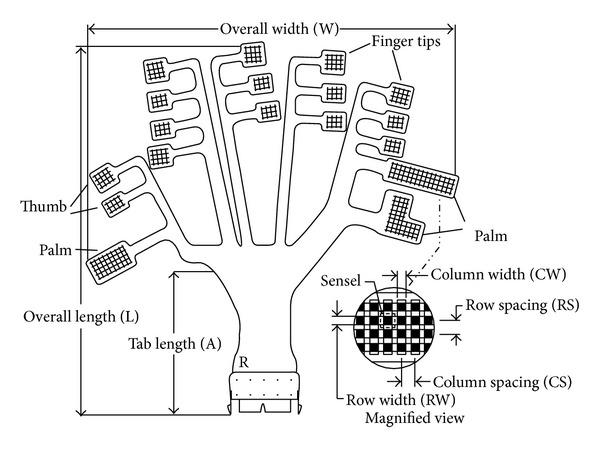
The* Grip* sensors prior to attachment to a vinyl glove [27].

The* Grip* system includes a series of force transducers mounted on a flexible force plate which can mould to the shape of the hand. Each force transducer (sensel) consists of a unique piezoresistive material sandwiched between two pieces of flexible polyester, with printed silver conductors on each half [27]. Software is provided which can generate force data or force mapping of the contact area. The* Grip* has 18 sensing regions that are positioned over the fingers and palm (Figure 3). Gaps between the sensing areas allow the joints to move freely and not interfere with grip measurement. Each sensing region has multiple sensels for localized identification of contact points on the hand. There are a total of 349 sensels in the array and the sampling rate for the system is 30 Hz.

The study was conducted at an indoor circuit at the University of Brighton (Figure 5). All participants were given familiarisation training in the use of both the wheelchairs until they felt competent to undertake the trial. The steering for both wheelchairs involved the Neater Uni-wheelchair steering mechanism. Maneuvering the Neater Uni-wheelchair involved the use of the single rim which was attached to the rear wheel differential for propulsion. Maneuvering the foot steered Action3 involved propulsion only using the single handrim on one side of the wheelchair.

The total length of the driving course in the indoor circuit was 150 m. The distance was standardised for all participants and the time taken to complete each activity within the course was recorded. Participants were initially asked to drive across the gymnasium floor for 30 m, complete a 90° left turn, and continue for 10 m. A further 90° left hand turn took the user onto carpet and brush matting. The carpet and matting were 30 m long. At the end of the carpet the user made a 135° left hand turn into a series of corners consisting of three closely placed bollard markers which required tight 45° right- and left-hand turns. At the end of the corners, the user completed a 135° right-hand turn for a further 30 m of straight driving to take the user back to the start/finish line.

### 3.2. Procedure

Demographic data including age and gender were recorded for all subjects. The users hand was measured for size using a preinstrumented vinyl glove to which the Grip sensors had been attached (Figure 6) according to the manufacturer instructions [27].

The user's right hand was dusted with talcum powder prior to putting on a first tight fitting noninstrumented vinyl glove. This vinyl glove was also dusted with talcum powder and then inserted into the preinstrumented vinyl glove (Figure 6). The procedure of double gloving was for hygiene reasons. The use of talcum powder facilitated easy removal of the instrumented glove without damage to the sensors.

The preinstrumented glove was attached to the VersaTek cuffs (Figure 7) which processed the data and relayed it to the computer via USB connection.

The system was calibrated for each subject prior to data collection as recommended by the manufacturer [27]. Subjects were randomly allocated the wheelchairs using random numbers.

The participants were asked to drive each wheelchair round the course (Figure 5). Data was captured continuously throughout each circuit. Time taken to complete each activity within the circuit was recorded. The activities were defined as straight running, mats (simulating resistance), and corners.

The key time points were

1. A-B: start and straight running to first bend;
2. C-D: beginning of mats to end of mats and third bend;
3. D-E: beginning of the corner to final bend.

The course was repeated once per wheelchair with a 30 minute gap, or however much time was necessary, for the users to feel recovered.

### 3.3. Data Processing

The force time data for each region was exported into Excel. A linear trapezoidal integration of force was performed for each region. The total grip force generated at the hand/handrim interface for each activity (straight running, corners, and mats) was recorded.

**Figure 4:**
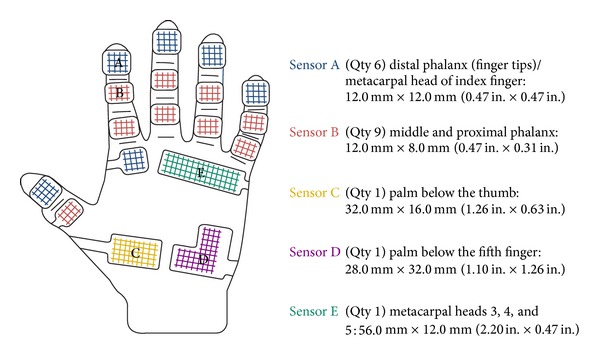
Location of the sensors after attachment to a glove [27].

**Figure 5:**
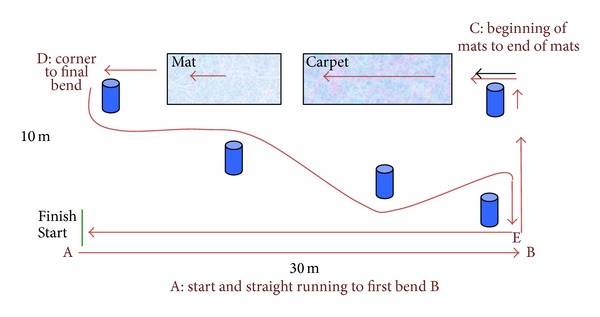
The driving course.

**Figure 6:**
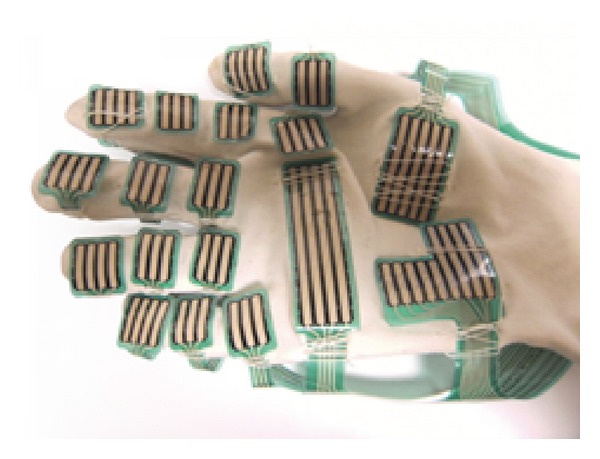
The preinstrumented glove showing the Grip attached to the vinyl glove [27]. Photographs courtesy of SATRA Technology Centre.

**Figure 7:**
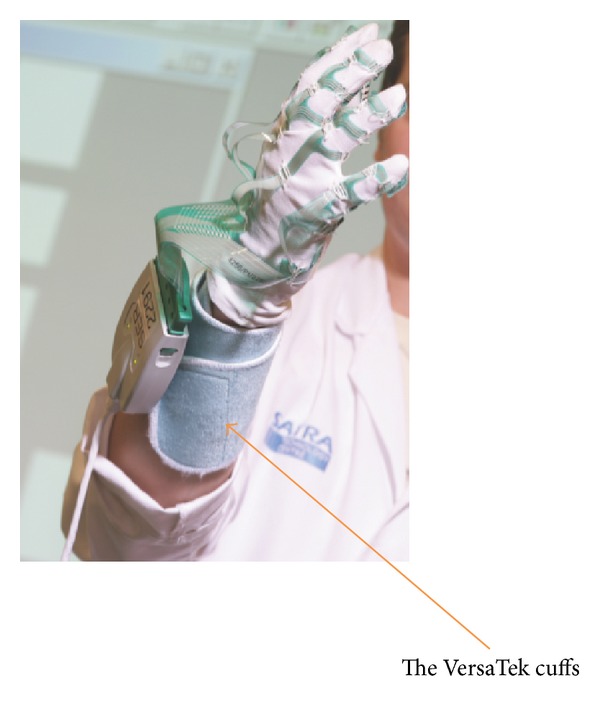
The VersaTek cuffs [27]. Photographs courtesy of SATRA Technology Centre.

The raw grip force data was also manipulated using the* Grip* software to generate three regions of the hand consisting of thumb, digit, and palm fields.  Figure 4 shows the position of the sensors. The thumb field consisted of two sensors, the palm field 4 sensors, and the finger field 3 sensors/finger, totaling 12 sensors.

**Figure 8:**
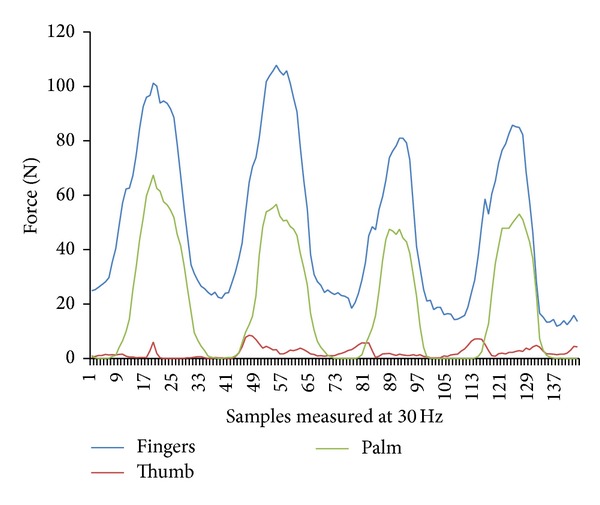
An example of raw data from the finger, thumb, and palm fields during straight running propulsion in the NUW.

Figure 8 shows an example of the grip forces generated in the finger, thumb, and palm field during straight running propulsion in the NUW.

For each field the data from all sensors were totaled. Totals were calculated for each of the three fields for each activity (straight running, mats, and corners) in each wheelchair.

### 3.4. Statistical Analysis

The data were investigated to explore the differences in forces in the different regions of the hand, over different surfaces between the two different wheelchairs across the whole sample. The data was not normally distributed and was logarithmically transformed using a log⁡⁡10 manipulation to enable a three-way ANOVA with Tukey's post hoc test (*α* < 0.05) to be undertaken to explore the forces across the regions of the hand. The three independent variables were type of wheelchair, activity, and region of the hand. The total forces were analysed using a two-way ANOVA to explore the differences between the wheelchairs.

The two independent variables were wheelchair and activity.

## 4. Results

The gender distribution was 10 women and 7 men (Table 1).

There was no difference between the time taken to complete each activity within the circuit in the different wheelchairs (*P* = 0.245). The mean (SD) time taken to complete the straight running section of the course was 13.54 (3.86) secs in the Neater Uni-wheelchair and 12.94 (3.56) secs in the Action3 wheelchair. This would indicate that the velocity in straight running would be 0.71 (0.19) m/sec in the Neater Uni-wheelchair and 0.74 (0.18) m/sec in the Action3 wheelchair. This was also shown not to be significant (*P* = 0.548). The time taken to complete the corners section of the course was 11.76 (3.03) secs in the Neater Uni-wheelchair and 11.76 (3.93) secs in the Action3 wheelchair. For the mats section of the course the times were 19.12 (4.04) secs for the Neater Uni-wheelchair and 19.41 (4.64) secs for the Action3 wheelchair. These results were also not significant.

Total grip force and hand component grip forces are described in Table 2.

### 4.1. Total Handgrip Forces

The results of the 3-way ANOVA showed that there was a significant difference between the forces generated in propelling the different wheelchairs (*F* = 5.016,  *df* = 1, and  *P* = 0.027).

Post hoc* t*-tests indicated that there were significant differences between the wheelchairs in straight running (*t* = −2.729,  *df* = 16,  and *P* = 0.015). Greater total force was exerted when propelling the foot steered Action3 in straight running. There were no significant differences in force generation between the wheelchairs around corners (*t* = −0.719,  *df* = 16, and *P* = 0.483) or over mats (*t* = −1.463,  *df* = 16, and *P* = 0.163).

### 4.2. Grip Forces across the Different Regions of the Hand

The results of the 3-way ANOVA showed that there were significant differences in the force exerted between the regions (*F* = 168.428,  *df* = 2, and *P* < 0.001) and the wheelchairs (*F* = 4.782, *df* = 1, and *P* < 0.03).

Post hoc* t*-test suggested that there were significant differences between the wheelchairs in force exerted through the fingers (*t* = −2.634, *P* = 0.018) and thumbs (*t* = −3.301, *P* = 0.005) in straight running. Greater forces were exerted in propelling the foot steered Action3 wheelchair.

There were no significant differences between the wheelchairs in any of the other activities.

Post hoc Tukey's test to explore differences between regions of the hand demonstrated differences between all regions with the fingers generating a greater force than the palm (*M* = 0.5029, 95% CI [0.4156,0.5902], and *P* < 0.001) and thumb (*M* = 0.648, 95% CI [0.5607,0.7353], and *P* < 0.001). The palm generated more force than the thumb (*M* = 0.1452, 95% CI [0.0579,0.2325], and *P* < 0.001).

## 5. Discussion

The aim of this study was to measure and compare the total and regional handgrip forces measured at the hand/handrim interface during propulsion, in a sample of nondisabled participants using right-sided one arm drive wheelchairs. The objective of the study was to explore the grip forces generated when maneuvering different one arm drive wheelchairs in a controlled environment and around obstacles. The work is novel because grip forces have not been measured in any one arm drive wheelchairs.

The results indicated that users applied higher total grip forces and finger and thumb forces at the hand/handrim interface in straight running when using the foot steered Action3 wheelchair when compared to the Neater Uni-wheelchair. This difference may be explained through the action of the differential in the Neater Uni-wheelchair. The differential ensures that the force applied to the handrim delivers equal torque to both drive wheels of the wheelchair, thus ensuring that the wheelchair will be propelled more easily in a straight line. In the foot steered Action3 wheelchair, the force is applied to only one rear wheel. This results in a turning moment with the wheelchair tending to turn away from a straight path. To compensate for this, the front steering wheel would need to be maintained to apply an equal and opposite turning moment to keep the wheelchair path straight. Depending upon the nature of the surface, the propelling force acting on the turned steering front wheel could result in an increase in forward rolling resistance. This would be sufficient to explain the differences found in total force.

In light of the results from straight running, it was speculated that the results for the force during propulsion over the mats would also be lower in the Neater Uni-wheelchair. However, this was found to not be the case, although it was observed that during the data collection all the users struggled to manoeuvre each of the wheelchairs over the mats. This may suggest that if the differences in hand/handrim grip force were due to increased rolling resistance resulting from the compensatory turned position of the front steered wheel, this effect may have been masked by the greater effort required to propel the wheelchairs over the resistive surfaces. Further work could explore this in greater detail.

Early studies have investigated the influence of handle diameter on wheelchair propulsion rather than hand/handrim force [28]. Recent studies [24, 25] used the* Grip* measure tool to measure hand interface force in a variety of handle diameters. Their findings concur with earlier work [28] that a larger diameter handrim requires greater force for propulsion, reporting that the optimum diameter to develop the maximal handgrip force is between 20 and 30 mm. In this study the handrims in both the foot steered Action3 and the Neater Uni-wheelchair were the standard manufacturer handrims (20 mm) and within the optimum range.

The results from the two wheelchairs could also have been influenced by differences in the weights of the chairs, their rolling resistances, and the velocity with which they were propelled. In this study the two wheelchairs were both of the same model and both had the same foot steering device attached. The only difference between the wheelchairs was the addition of the differential to the Neater Uni-wheelchair. The difference in weight between the wheelchairs was negligible (0.1 kg) which theoretically would suggest that there would be no difference in rolling resistance. It is, however, acknowledged that rolling resistance was not measured in the study. Moreover, the velocity of wheelchair propulsion was also not controlled in this study. However, as the time taken to complete the circuits did not vary significantly, it could be suggested that the velocity would also not have varied. Greater control of the velocity using a treadmill would eliminate this variable from the study; however this in turn would have precluded simulation of functional use of the wheelchair.

The results from the analysis of the regions of the hand also provided some interesting findings which supported those of the total grip force analysis. The results suggested that forces generated in the fingers and thumbs were greatest in the foot steered Action3 wheelchair in straight running. These findings concur with the results reported by Medola et al. [25] who showed that, when propelling a wheelchair, the hands hold the handrim with the distal phalanges of the fingers, concentrating higher force on these areas. The increase in grip forces generated by the fingers would indicate that a stronger grip was being applied when propelling the foot steered Action3 wheelchair. There was no significant difference in hand/handrim forces when propelling the wheelchairs over the mats or around corners.

The evidence suggests that, while it is possible to modify a standard Action3 wheelchair for use by people with use of one arm by attaching an independent steering mechanism, the grip force exerted at the hand/handrim interface will be significantly greater in straight running than the wheelchair with both components of the Neater Uni-wheelchair kit. Whilst this may be statistically significant, further work is required to establish if this has any clinical significance within a user population.

## 6. Conclusion

The measurements of grip forces have only previously been reported in standard manual wheelchairs and not in manual one arm drive wheelchairs. The findings from this novel study, whilst concurring with findings from the standard wheelchair literature, add to our understanding of handgrip forces exerted in one arm drive wheelchairs. The results suggest that the Action3 with foot steering generated greater grip forces which may infer a greater potential for repetitive strain injury in the upper limb. Modifying an Action3 wheelchair with the addition of a steering mechanism may be a potentially more economical alternative to the Neater Uni-wheelchair, but one which may not be so ergonomically efficient. Further work is required to explore whether the difference in grip force is of clinical significance in a disabled population.

## Figures and Tables

**Table 1:** To show demographic variables of the participants.

Male	Mean	SD	Female	Mean	SD
Age (yrs)	25.86	11.05	Age (yrs)	30.3	11.34
Height (cm)	183	9.70	Height (cm)	166.9	6.54
Weight (kg)	77.29	19.03	Weight (kg)	62.1	7.43

**Table 2:** Descriptive log transformed data to show mean and standard deviation of force measurement (*N*) per region per wheelchair for each activity.

Region	Wheelchair	Straight running mean (SD)	Corners mean (SD)	Mats mean (SD)
Fingers	Action3	2.81∗ (0.18)	2.90 (0.35)	2.76 (0.24)
	Neater	2.71 (0.17)	2.85 (0.17)	2.67 (0.23)

Thumb	Action3	2.22∗ (0.18)	2.26 (0.35)	2.09 (0.28)
	Neater	2.10 (0.19)	2.17 (0.18)	1.97 (0.23)

Palm	Action3	2.26 (0.31)	2.30 (0.45)	2.28 (0.31)
	Neater	2.30 (0.27)	2.33 (0.25)	2.21 (0.25)

Total	Action3	7.29∗ (0.56)	7.47 (1.09)	7.13 (0.78)
	Neater	7.12 (0.52)	7.34 (0.54)	6.85 (0.68)

Key

∗signifies significant difference between the two wheelchairs.
